# In Vitro Interaction of Organophosphono- and Organophosphorothioates with Human Acetylcholinesterase

**DOI:** 10.3390/molecules25133029

**Published:** 2020-07-02

**Authors:** Franz Worek, Horst Thiermann, Marianne Koller, Timo Wille

**Affiliations:** Bundeswehr Institute of Pharmacology and Toxicology, D-80937 Munich, Germany; horstthiermann@bundeswehr.org (H.T.); mariannekoller@bundeswehr.org (M.K.); timowille@bundeswehr.org (T.W.)

**Keywords:** organophosphorus compounds, nerve agents, analogues, acetylcholinesterase, in vitro kinetics, structure–activity relationship

## Abstract

The implementation of the Chemical Weapons Convention (CWC) in 1997 was a milestone in the prohibition of chemical warfare agents (CWA). Yet, the repeated use of CWA underlines the ongoing threat to the population. Organophosphorus (OP) nerve agents still represent the most toxic CWA subgroup. Defensive research on nerve agents is mainly focused on the “classical five”, namely tabun, sarin, soman, cyclosarin and VX, although Schedule 1 of the CWC covers an unforeseeable number of homologues. Likewise, an uncounted number of OP pesticides have been produced in previous decades. Our aim was to determine the in vitro inhibition kinetics of selected organophosphono- and organophosphorothioates with human AChE, as well as hydrolysis of the agents in human plasma and reactivation of inhibited AChE, in order to derive potential structure–activity relationships. The investigation of the interactions of selected OP compounds belonging to schedule 1 (V-agents) and schedule 2 (amiton) of the CWC with human AChE revealed distinct structural effects of the *P*-alkyl, *P*-*O*-alkyl and *N*,*N*-dialkyl residues on the inhibitory potency of the agents. Irrespective of structural modifications, all tested V-agents presented as highly potent AChE inhibitors. The high stability of the tested agents in human plasma will most likely result in long-lasting poisoning in vivo, having relevant consequences for the treatment regimen. In conclusion, the results of this study emphasize the need to investigate the biological effects of nerve agent analogues in order to assess the efficacy of available medical countermeasures.

## 1. Introduction

The implementation of the Chemical Weapons Convention (CWC) in 1997 [1] was a milestone in the prohibition of chemical warfare agents (CWA). However, the repeated use of CWA in military conflicts such as in Syria, by terrorists and against individuals, underlines the ongoing threat to the population [2,3,4,5] and demands further research on the biological properties of CWA and improved medical countermeasures against these agents.

Organophosphorus (OP) nerve agents still represent the most toxic CWA subgroup, with their toxicity being a result of the covalent inhibition of the pivotal enzyme acetylcholinesterase (AChE) that induces an overstimulation of cholinergic receptors and eventually leads to death by respiratory arrest [5,6]. Defensive research on nerve agents is mainly focused on the “classical five”, namely tabun, sarin, soman, cyclosarin and VX, although Schedule 1 of the CWC covers an unforeseeable number of homologues. Likewise, an uncounted number of OP pesticides have been produced in the previous decades [7,8].

Various studies have dealt with structure–activity relationships of OP nerve agents and pesticides, mainly focusing on the in vitro and in vivo toxicity of structural analogues [9,10,11,12,13,14,15,16,17,18]. Our group investigated the interaction of sarin and tabun analogues with human AChE and butyrylcholinesterase (BChE). We determined the inhibition, aging and reactivation kinetics and gave an insight into the impact of different OP residues on kinetic parameters [19,20,21,22].

**Figure 1:**
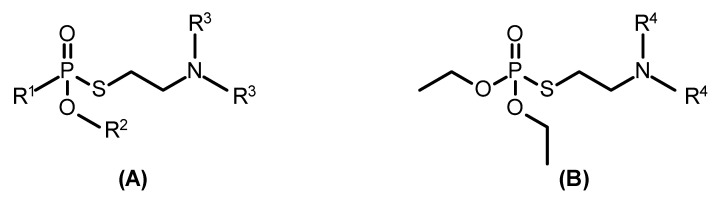
Generic chemical structure of organophosphonothioates (**A**) and organophosphorothioates (**B**).

In light of these previous findings, it was tempting to have a closer look at the in vitro inhibition kinetics of selected organophosphono- (Figure 1A) and organophosphorothioates (Figure 1B) with human AChE, as well as hydrolysis of the agents in human plasma and reactivation of inhibited AChE, in order to derive potential structure–activity relationships.

## 2. Results

The investigation of the inhibition kinetics of organophosphonothioates (Figure 1) with human AChE revealed a partial structure–activity relationship. With agents bearing *P*-methyl and *O*-ethyl residues, the inhibitory potency increased with varying *N*,*N*-dialkyl groups in the order methyl (**1**) < ethyl (**2**) < *n*-propyl (**3**) < *i*-propyl (**4**) (Table 1). A comparable relationship was recorded for agents bearing *P*-methyl and *N*,*N*-diethyl residues and varying *O*-alkyl groups, i.e., methyl (**5**) < ethyl (**2**) < *n*-butyl (**6**) < *i*-butyl (**7**) (Table 1). A comparison of agents with identical *N*,*N*-dialkyl groups but different *P*-alkyl residues showed a higher inhibition rate constant with compounds bearing a *P*-methyl compared to *P*-ethyl group (Table 1). No obvious relationship was observed with compounds **8**, **9** and **10**.

With a small series of organophosphorothioates, amiton analogues, an increase of inhibitory potency in relation to the *N*,*N*-dialkyl residue was determined, i.e., an increase in the order methyl (**11**) < ethyl (**12**) < *i*-propyl (**13**) (Table 2).

The stability of the test compounds in human heparin plasma was determined with an AChE inhibition assay. It turned out that agents bearing a *P*-methyl group but variable *O*-alkyl and *N*,*N*-dialkyl residues (**1**–**7**) had a comparable degradation half-time in the range of 17 to 39 h (Table 1). The degradation velocity was substantially lower, i.e., ~100 h, with agents bearing a *P*-ethyl group (Table 2). Interestingly, *N*,*N*-dialkyl residues had a substantial impact on the degradation half-time of amiton derivatives (Table 2), which increased in the order *i*-propyl (**13**) < ethyl (**12**) < methyl (**11**) and reached almost 200 h with agent **11**.

**Figure 2:**
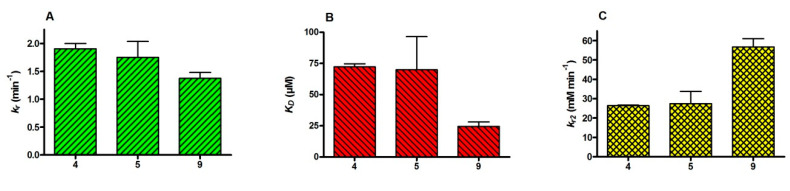
Reactivation kinetics of obidoxime with human AChE inhibited by compounds **4**, **5** and **9** (cf. Table 1). Data are shown as means ± SD with (**A**) reactivity constant *k_r_*, (**B**) dissociation constant *K_D_* and (**C**) bimolecular reactivation rate constant *k_r2_*.

The effect of different residues at the central phosphorus on the reactivation of inhibited human AChE by obidoxime was tested with **4**, **5** and **9** by the determination of reactivation rate constants. It turned out that the different residues had only a small impact on the reactivity rate constant (Figure 2A), but obidoxime had a substantially higher affinity with AChE inhibited by **9** (Figure 2B), leading to a 2-fold higher bimolecular reactivation rate constant compared to **4** and **5** (Figure 2C).

## 3. Discussion

The determination of the inhibitory potency of the tested organophosphonothioates revealed a major impact of the *O*-alkyl residues, with a more than 20-fold increase of *k_i_* values comparing *O*-methyl and *O*-*i*-butyl (Table 1). This corresponds to data from previous studies with a series of methylphosphonofluoridates showing an almost 90-fold difference between *O*-*n*-pentyl and *O*-methyl [19,21] and with a study testing a homologous series of organophosphonothioates bearing a *N*,*N*-dimethyl residue [11]. The influence of the *N*,*N*-dialkyl residues on the inhibitory potency was less pronounced, leading to a 5-fold increase from methyl to *i*-propyl (Table 1). Bajgar and Patocka observed an ~10-fold increase of the inhibition rate constants from methyl to *i*-propyl with human brain AChE, and Hall and co-workers also recorded an ~5-fold increase of the inhibition rate constant from *n*-propyl to *n*-pentyl using bovine erythrocyte AChE [13,23]. With amiton analogues, *N*,*N*-dialkyl residues had a moderate influence on the inhibitory potency, with an ~3-fold increase from methyl to *i*-propyl (Table 2). Interestingly, with **8**, **9** and **10**, bearing a *P*-ethyl and *O*-ethyl group, the *N*,*N*-dialkyl residues were of less impact on *k_i_* values. Finally, in pairs of agents with identical *O*-alkyl and *N*,*N*-dialkyl residues, *P*-methyl showed a higher inhibitory potency compared to *P*-ethyl (Table 1), which may indicate that asymmetric OP compounds are more potent AChE inhibitors than their symmetrical analogues [24].

To this end, the data of the present study as well as previous work demonstrate an obvious positive relationship between the size of the *O*-alkyl residue, as well as of the *N*,*N*-dialkyl residues and the inhibitory potency of an OP towards AChE. However, in vivo studies with different animal species indicate an at least partial discrepancy between in vitro inhibitory potency and in vivo LD_50_ values, underlining the need for validation of in vitro data by appropriate animal experiments [13,15].

In comparison to G-type nerve agents such as tabun, sarin or cyclosarin, VX and its close analogues are rather resistant towards chemical and enzymatic hydrolysis [25,26,27]. Accordingly, a previous study with human fresh frozen plasma revealed a degradation half-time of 5 to 30 min with tabun, sarin, cyclosarin and soman while a t½ of ~30 h was recorded for VX, VR and CVX [28]. By using fresh heparinized human plasma, rather comparable results were found with agents **1**–**7**, all bearing a *P*-methyl group (Table 1). In contrast, replacement of *P*-methyl by *P*-ethyl resulted in a markedly higher stability and resulted in a degradation half-time in plasma of ~100 h. Likewise, degradation of amiton analogues in plasma was slow, with a half-time between 195 h (methyl; **11**) and 64 h (*i*-propyl; **13**) (Table 2). Results from a previous study showed a substantial acceleration of V-agent degradation in the presence of plasma in comparison to spontaneous degradation in buffer. This process was not affected by the addition of the calcium chelator and inhibitor of paraoxonase-mediated hydrolysis EDTA and aurin tricarboxylic acid ammonium salt (aluminon), an inhibitor of albumin-mediatiated hydrolysis [29], but followed first-order kinetics [28]. In the present study, incubation of all tested agents in plasma resulted in a marked acceleration of degradation in comparison to incubation in buffer. The underlying mechanism of this presumably catalytic process is presently unknown.

Previous animal studies demonstrated a long persistence of VX and VR in the systemic circulation [30,31]. This implicates that exposure to any of the tested compounds will most likely result in long-lasting, toxicologically relevant agent concentrations and will require prolonged (intensive care) treatment [32].

Human AChE inhibited by the main representative of V-agents, VX, can be easily reactivated by obidoxime, while derivatives bearing a bulkier *O*-alkyl group, i.e., VR and CVX, are less susceptible to reactivation [33,34]. In fact, the determination of reactivation kinetics of obidoxime with human AChE, inhibited by a homologous series of methylphosphonofluoridates, revealed a decrease of the bimolecular reactivation rate constant with increasing size of the *O*-alkyl residue (methyl to pentyl/cyclohexyl) [19,21]. Hence, in the present study, reactivation experiments were only undertaken with three agents bearing variable *P*-alkyl and *O*-alkyl groups, i.e., methyl/methyl (**5**), methyl/ethyl (**4**) and ethyl/ethyl (**9**) (Table 1). There was virtually no difference between **4** and **5** regarding affinity and reactivity of obidoxime, while with **9** the oxime had a markedly higher affinity resulting in a 2-fold higher bimolecular reactivation rate constant (Figure 2). It appears that the slightly bulkier *P*-ethyl group facilitates the access of obidoxime to the phosphyl residue. Further studies with agents bearing *P*-ethyl and variable *O*-alkyl residues are needed to gain more insight into the reactivation mechanism.

In conclusion, the investigation of interactions of selected OP compounds belonging to Schedule 1 (V-agents) and Schedule 2 (amiton) of the Chemical Weapons Convention with human AChE revealed distinct structural effects on the inhibitory potency of the agents. Irrespective of structural modifications, all tested V-agents presented as highly potent AChE inhibitors. Amiton and its two analogues were less potent than V-agents but still better AChE inhibitors than most of the marketed OP pesticides. The high, in part extraordinary, stability of the tested agents in human plasma will most likely result in long-lasting poisoning in vivo. This will have relevant consequences for the treatment regimen and will pose a burden on medical resources.

## 4. Materials and Methods 

### 4.1. Materials

Organophosphorus compounds (>98% by GC–MS, ^1^H NMR and ^31^P NMR) were made available by the German Ministry of Defence. Obidoxime dichloride was purchased from Ferak Chemie (Berlin, Germany). Acetylthiocholine iodide (ATCh) and 5,5′-dithiobis(2-nitrobenzoic acid) (DTNB) were supplied by Sigma-Aldrich (Taufkirchen, Germany). All other chemicals were from Merck (Darmstadt, Germany).

OP stock solutions (0.1% *v*/*v*) were prepared in acetonitrile and were stored at room temperature. Oxime stock solutions (200 mM) were prepared in distilled water and were stored at −80 °C. Working solutions were appropriately diluted in distilled water just before the experiment and were kept on ice until use.

Hemoglobin-free human erythrocyte ghosts were prepared as described from heparinized human blood and served as AChE source [35]. Aliquots of the erythrocyte ghosts with an AChE activity adjusted to that found in whole blood were stored at –80 °C, and aliquots were homogenized prior to use to achieve a homogeneous matrix for the kinetic studies.

### 4.2. AChE Activity

AChE activities were measured with a modified Ellman assay [36,37] at 412 nm (Cary 50 Bio, Varian, Darmstadt, Germany) using polystyrol cuvettes, 0.45 mM ATCh as substrate and 0.3 mM DTNB as a chromogen in 0.1 M phosphate buffer (pH 7.4).

All experiments were performed at 37 °C and pH 7.4. All concentrations refer to final concentrations.

### 4.3. Inhibition Kinetics

The OP inhibition kinetics were determined in the presence of substrate as described before [33]. In brief, 10 µL erythrocyte ghosts and 5 µL diluted OP (8 different concentrations) were added to a cuvette containing phosphate buffer, DTNB and ATCh (final volume 3.165 mL). ATCh hydrolysis was continuously monitored for 5 min. The recorded curves were analyzed by nonlinear regression analysis and used for the further determination of the bimolecular inhibition rate constant *k_i_* [38]. All experiments were performed in duplicate.

### 4.4. Reactivation Kinetics

The reactivation rate constants of obidoxime were determined by a continuous procedure [34]. Here, 10 µL OP-inhibited AChE was added to a cuvette containing phosphate buffer, DTNB, ATCh and specified oxime concentrations (final volume 3.16 mL). ATCh hydrolysis was continuously monitored over 5 min. Activities were individually corrected for oxime-induced substrate hydrolysis. The final oxime concentration during this assay was limited to 100 µM obidoxime. Eight to ten different oxime concentrations were used for the determination of the reactivation rate constants in duplicate.

The constants *K_D_*, which approximates the dissociation constant and is inversely proportional to the affinity of the oxime for the inhibited enzyme, and *k_r_*, indicating the reactivity of the oxime, were calculated as described before [34]. The bimolecular reactivation rate constant *k_r2_* was calculated from the ratio of *k_r_* and *K_D_*.

### 4.5. OP Stability in Plasma

The degradation kinetics of agents **1**–**13** (Table 1 and Table 2) in heparinized human plasma were investigated by an AChE inhibition assay as described before [28]. In brief, 500 µL TRIS (200 mM + 2 mM CaCl_2_; pH 7.4) was mixed with 500 µL plasma and incubated at 37 °C. Then, 5 µL agent was added (t = 0). At defined time points (0, 2, 6, 24, 48, 72 and 96 h), 30 µL samples were taken and added to a prewarmed (37 °C) polystyrol cuvette that previously had been filled with 3 mL phosphate buffer (0.1 M; pH 7.4), 100 µL DTNB (0.3 mM final concentration) and 50 µL ATCh (0.45 mM final concentration). Finally, 10 µL AChE was added and the AChE activity was measured spectrophotometrically with the modified Ellman assay for 5 min at 412 nm. The recorded AChE inhibition curves were analyzed by nonlinear regression analysis to determine the first-order inhibition rate constant *k_1_,* which was corrected for the spontaneous agent hydrolysis. Then, *k_1_* was plotted against time to calculate the degradation half-time.

### 4.6. Data Analysis

Processing of experimental data for the determination of the different kinetic constants was performed by nonlinear regression analysis using curve fitting programs provided by Prism Version 4.03 (GraphPad Software, San Diego, CA, USA).

## Figures and Tables

**Table 1:** Chemical structure of tested organophosphonothioates, AChE inhibition kinetics (k_i_) and stability in human plasma (t_½_).

Agent	R^1^	R^2^	R^3^	*k*_i_ (10^7^ M^−1^ min^−1^)	t_½_ (h)
**1**	Methyl	Ethyl	Methyl	2.2 ± 0.18	39.2 ± 1.0
**2**	Methyl	Ethyl	Ethyl	5.5 ± 0.25	35.3 ± 0.6
**3**	Methyl	Ethyl	*n*-Propyl	7.9 ± 0.67	29.4 ± 0.8
**4**	Methyl	Ethyl	*i*-Propyl	11 ± 0.8	31.7 ± 0.2
**5**	Methyl	Methyl	Ethyl	1.9 ± 0.19	16.9 ± 1.7
**6**	Methyl	*n*-Butyl	Ethyl	32 ± 1.0	20.3 ± 1.2
**7**	Methyl	*i*-Butyl	Ethyl	46 ± 0.2	35.7 ± 5.5
**8**	Ethyl	Ethyl	Methyl	1.3 ± 0.31	108.1 ± 0.5
**9**	Ethyl	Ethyl	Ethyl	4.3 ± 0.07	93.9 ± 2.1
**10**	Ethyl	Ethyl	*i*-Propyl	3.7 ± 0.13	98.9 ± 7.4

R^1^, R^2^ and R^3^ refer to Figure 1A.

**Table 2:** Chemical structure of tested organophosphorothioates, AChE inhibition kinetics (*k*_i_) and stability in human plasma (t_½_).

Agent	R^4^	*k*_i_ (10^5^ M^−1^ min^−1^)	t_½_ (h)
**11**	Methyl	8.6 ± 0.10	194.5 ± 18.3
**12**	Ethyl	19 ± 0.7	152.5 ± 0.3
**13**	*i*-Propyl	27 ± 0.2	63.6 ± 1.3

R^4^ refers to Figure 1B.
